# Geriatric Assessment in Multicultural Immigrant Populations

**DOI:** 10.3390/geriatrics4030040

**Published:** 2019-06-26

**Authors:** Katherine T. Ward, Mailee Hess, Shirley Wu

**Affiliations:** 1 Section of Geriatrics, Division of General Internal Medicine, Department of Medicine, Harbor-UCLA Medical Center, Torrance, CA 90509, USA; 2 David Geffen School of Medicine at UCLA, Los Angeles, CA 90095, USA

**Keywords:** multicultural, geriatric assessment, ethnogeriatrics, immigrant, social determinants

## Abstract

While the traditional comprehensive geriatric assessment provides valuable information essential to caring for older adults, it often falls short in multicultural immigrant populations. The number of foreign-born older adults is growing, and in some regions of the United States of America (U.S.), they encompass a significant portion of the older adult population. To ensure we are caring for this culturally diverse population adequately, we need to develop a more culturally competent comprehensive geriatric assessment. In this review, we explore ways in which to do this, address areas unique to multicultural immigrant populations, and identify limitations of the current assessment tools when applied to these populations. In order to be more culturally sensitive, we should incorporate the concepts of ethnogeriatrics into a comprehensive geriatric assessment, by addressing topics like healthcare disparities, language barriers, health literacy, acculturation level, and culturally defined beliefs. Additionally, we must be sensitive to the limitations of our current assessment tools and consider how we can expand our assessment toolkit to address these limitations. We discuss the limitations in cognitive screening tests, delirium assessments, functional and mental health assessments, advance care planning, and elder abuse.

## 1. Introduction: Geriatric Assessment in Multicultural Immigrant Populations

The comprehensive geriatric assessment has proven to be a valuable tool to address the health and wellness of the older adult population. However, geriatric assessment tools were developed within Western biomedical models that may not apply to all subpopulations of older adults in the United States of America (U.S.). In this paper, we seek to review and recommend best practices on approaching geriatric assessment in a multicultural immigrant population. We explore the challenges that need to be considered for the geriatric assessment in a multicultural immigrant population, the evidence that highlights the inadequacy of our current assessment, and some potential solutions to improve our geriatric assessments.

In 2001, the Institute of Medicine published “Retooling for an Aging America,” describing the need for a health care workforce that can address the diversity of the aging population in the U.S. and suggesting that the health of individuals depends on more than just their vital signs, laboratory tests, or physical examinations; individual health is influenced by culture, language, national origin, religion, education, income and assets, level of acculturation, and more [1]. Addressing these issues requires that policy-makers and health care providers be familiar with the individual patient’s health beliefs, risk for diseases, family systems, access to care, and dependency on adult children.

The U.S. population encompasses approximately 50 million immigrants, and census data suggest that 15% of adults 60 years and older are foreign-born [2]. This population is expected to increase; the proportion of foreign-born older adults in 2030 is estimated to be 25%, increasing to 35% by 2050 [3]. The foreign-born population is not geographically uniform. In California, foreign-born older adults comprise more than a third of the older adult population; in Los Angeles County, this population approaches 50%. A more culturally sensitive approach to geriatrics is particularly important in these communities. 

Undocumented older adult immigrants have additional needs, and this population is expected to grow rapidly over the next 15 years. Eleven million immigrants in the U.S. are undocumented, of which 10% are currently older than 55. Undocumented older adult immigrants face additional challenges beyond serious chronic health issues, including cognitive disorders and physical injuries with functional impairment. They may work and pay into Social Security and Medicare, yet are unable to claim these benefits as they age due to their undocumented status. They often have poor access to health care and do not receive primary preventive care, which results in more expensive care [3]. Furthermore, they are also at increased risk for elder abuse; their immigration status and fear of deportation is a vulnerability that can be exploited [4,5]. 

## 2. Challenges to the Geriatric Evaluation in a Multicultural Immigrant Population

While cross-cultural health care has been taught in health professions training programs since the 1970s, it was not until the 1990s that it was adapted to geriatric training programs. Around that time, the term “ethnogeriatrics” emerged and was defined as culturally competent health care for older adults [6]. A curricular framework for multicultural geriatric care was created and intended to be adaptable to various academic programs. The curriculum includes, for example, the effects of health care disparities, language, health literacy, acculturation level, and culturally defined beliefs [7]. Each of these aspects can have effects on the patient’s health directly but can also affect the provider’s assessment in areas of cognition, mood, and function. The topics of ethnogeriatrics are not typically included in the traditional comprehensive geriatric assessment, and yet are necessary to adequately care for immigrant older adult populations.

In addition, we also need better assessment instruments. The traditional comprehensive geriatric assessment tools have infrequently been validated in a multicultural immigrant geriatric population and, therefore, limit the provider’s ability to achieve high-quality multicultural care. For instance, cognitive tests are not validated for all languages, and most are not validated for populations with no formal education [8,9,10]. If an assessment tool has been studied in one ethnic group, it may not be applicable to a multicultural immigrant population as a whole. 

In Table 1, we summarize an approach to geriatric immigrant multicultural assessment and identify gaps that are opportunities for improvement. Gaps range widely depending on the existing literature. Several elements require the development of new assessment tools, others have existing tools that may need further validation in immigrant populations, and some need further downstream development and validation of interventions that improve outcomes in these populations. 

### 2.1. Health Care Disparities

Health care disparities are differences in health and health care among population groups. Health disparities and delayed health care among older adults have been found to be negatively associated with both self-reported health and mental health status. For example, population studies have found that foreign-born Asians and Latinos have the poorest self-reported health and mental health [11].

Due to the dramatic disparities in a multicultural immigrant population, the geriatric assessment should include an evaluation of which health conditions were delayed in diagnosis and treatment. For example, newly diagnosed diabetes needs to be treated, as well as the sequelae of delayed diagnosis, such as visual loss from diabetic retinopathy or diabetic kidney disease. 

Completion of preventive health care, such as vaccinations and cancer screening, is low in the immigrant older adult population [12]. Evidence-based guidelines do not address how to approach cancer screening in older adults who have passed the age of screening but who have not completed prior screenings. For example, do you screen for breast cancer in a 70-year-old woman with no prior breast cancer screenings? 

### 2.2. Language

The geriatric assessment should include identifying the preferred language of the patient and strategies for addressing communication barriers. 

Language barriers are an added complexity for a medical visit in any age group. While medical interpreters or telephone-based interpreter services are frequently utilized and mandated by The National Enhanced Culturally and Linguistically Appropriate Services Standards (CLAS) [13,14], using interpreters to communicate is time-consuming and an added challenge in a busy medical setting. 

Older adults have additional issues that can make communicating through interpreter services even more challenging, especially when using phone or video interpreters. Cognitive impairment and hearing impairment are frequently encountered in older adult populations and require additional time and patience [15]. If patients cannot hear the phone interpreter because of hearing impairment or do not understand how to use the interpreter because of cognitive impairment, family members are often relied upon to help with communicating with the patient. Although there may not be good alternatives, it is important to understand that this comes with risks. If family members do not speak English, a message will be repeated multiple times with a provider speaking to the interpreter who then speaks to the family member who then speaks to the patient. Alternatively, when family members do speak English, they often do not interpret directly but try to answer for the patient, resulting in the patient being removed from the conversation completely. Additionally, when family members are used to interpret, patients may refrain from discussing details or topics they do not want family members to know or to worry about. Although it has not been well studied, there is evidence that language barriers have some effect on patient safety and certainly can play a role in the provider’s ability to elicit patient symptoms, resulting in diagnostic errors [16]. 

Repetition using the “teach-back” method may improve information transfer [17]. When obtaining a history from a patient who does not speak the same language, summary statements and repeating back can help ensure accurate history-taking. Most interventions improving information transfer recommend the “teach-back” technique to ensure understanding. 

### 2.3. Health Literacy

Print literacy is the ability to read or write; health literacy is the degree to which individuals can understand, communicate, and act upon health information. Low health literacy can affect patients’ ability to follow through on a treatment plan, take medications correctly, or even understand what medical problems they have. Older adults, immigrants, minorities, and individuals with low incomes are more likely to have low health literacy. However, the inability to read and write or having a low educational level also contributes to low health literacy. The lack of understanding health information can theoretically lead to difficulty in engaging in health behaviors, preventative services, and disease management [18]. Health outcomes that have been measured related to low health literacy showed an increase in all-cause mortality rate [19] and a continual decline in baseline physical functioning [20]. 

While screening tools for health literacy exist, they are often impractical for the clinic setting. The concern for low health literacy is typically uncovered when working with patients directly. For instance, when discussing what they understand about their medical conditions or even obtaining a detailed history on whether they know when and which medicine to take can be enlightening. Documenting the concern for low health literacy can be helpful to communicate with other providers or specialists caring for the patient, but even more importantly, documenting possible solutions for addressing low health literacy can assist other providers to more effectively care for the patient [21]. Unstudied approaches to address health literacy include embedding community health workers (C.H.W.) in the team caring for the patient, or bringing the patient back for frequent nurse visits to manage chronic medical conditions such as hypertension and diabetes. When giving instructions about a treatment plan like how to take a medication, using teach-back methods can be helpful in ensuring patients understand what is being communicated. Additionally, involving other family members provides another layer of support. 

### 2.4. Acculturation Level

Acculturation is the dynamic process that commences when an immigrant enters into a new country and begins to adapt to its culture. The long-term consequences of the acculturation process are highly variable, difficult to study, and depend on social and personal variables of the society of origin and the society of settlement [22]. Studies have used different measures of acculturation, including language proficiency, leading to inconsistencies in the relationship between acculturation and health outcomes [23]. Self-reported health has a predictive role in clinical outcomes and mortality and is thought to be a reliable measure for monitoring population health [24]. One systematic review of nine studies showed an association between acculturation and fair to poor self-related health in non-white immigrants compared to Caucasians [25]. Longitudinal studies are needed that focus on measurements of self-related health at the time of immigration and across several years of the acculturation process.

### 2.5. Culturally Defined Beliefs

Three important culturally influenced beliefs should be assessed: (1) patients’ preferences for hearing health information and engaging in decision-making, (2) patients’ cultural perception of illness, and (3) the role of spirituality and religion in patients’ health [26]. Before these assessments, it is important to identify and respect older adults’ preferences for hearing health information and making their own health care decisions. For instance, do patients want to hear about illness directly from the provider or do they prefer that information is given to family first, who then decide what the patient should know? If the patients do not want to hear information about their illness, then they should not be their own primary healthcare decision-makers. There needs to be at least one person who can weigh the risks and benefits of treatments and diagnostic procedures and can therefore provide informed consent. Determining the appropriate decision-maker and respecting patients’ preferred communication parameters may help avoid communication breakdowns between providers and families. 

Patients’ perceptions of their illness and how they view the presentation of their illness is worth noting [27,28]. Using patients’ terminology and symptomatology can help bridge communication barriers. Additionally, understanding the importance of spirituality and religion for patients and how this shapes their view of their health can also be helpful. Spiritual advisors or native healers in the healthcare team should be included as appropriate [29,30]. 

## 3. Elements of the Geriatric Assessment

Validated tools in the typical assessment have limitations: they often do not account for cultural differences, language barriers, health literacy, or education level. Using these tools can lead to misdiagnosis of geriatric syndromes and missed opportunities to help older adults. While all areas of the geriatric assessment may benefit from a more culturally sensitive approach, in this section, we address the six main areas for which the most literature exists: cognitive impairment, delirium, mental health, function, advance care planning, and elder abuse. 

### 3.1. Cognitive Evaluation 

The number of immigrants with dementia is expected to rise [31]. Unfortunately, our commonly used tools for diagnosing dementia are problematic in populations with low English proficiency and low literacy. Brief cognitive tests are necessary to screen for or confirm a suspected dementia, yet many of the tests currently used have been shown to underestimate cognitive abilities in patients with low English proficiency and low literacy. This leads to an underestimation of the cognitive abilities of the patient and an overdiagnosis of cognitive impairment or dementia [8]. Other evidence suggests that using an interpreter for cognitive testing may significantly affect the scores for neuropsychological testing, further complicating the evaluation [32]. In a review of multiple different cognitive tests for suspected dementia, investigators found that the Rowland Universal Dementia Assessment Scale (RUDAS) performed the best in a low-English proficiency and low-literacy population. Furthermore, when compared to the Mini Mental Status Exam, the RUDAS had more diagnostic accuracy in a memory clinic population with very low education [33]. Until better information or validated tools specifically addressing this population become available, we recommend using the RUDAS for screening in populations with low education levels and with low English proficiency. 

### 3.2. Delirium Evaluation 

Additional care must be given for attention testing when evaluating for delirium. While tests like reciting the months of the year backwards or the days of the week backwards are reasonable attention tests in English [34], this is not true for all languages. In many languages, days of the week and months of the year are numbers instead of separate and distinct words. For example, in Chinese, the days of the week backwards would be 6, 5, 4, and so on; therefore, reciting them backwards is a fundamentally different task from reciting day names backwards, such as Sunday, Saturday, Friday, and so on. For languages that do not have distinct words for months or days, using a vigilance test, such as identifying letters or characters in a series, may be more appropriate. In the A vigilance test, the patient is instructed to squeeze the provider’s hand when they hear the letter A, then the provider spells out the phrase “S-A-V-E-A-H-A-A-R-T”. While the Confusion Assessment Method for the Intensive Care Unit (CAM-ICU) has been translated and validated in multiple languages which can be found on the Vanderbilt Critical Illness, Brain Dysfunction and Survivorship Center website, most of these tools are written and validated for a provider who reads and speaks the patient’s primary language and not necessarily for use with an interpreter [35].

### 3.3. Mental Health Evaluation

There are known disparities in adequate provider recognition of mental health issues due to language differences, health literacy barriers, and culturally specific presentations of distress. Furthermore, trained interpreters—even medical interpreters—may not be able to identify and interpret mental health issues during a geriatric evaluation. The level of acculturation and culturally defined beliefs can influence the mental health evaluation of a geriatric assessment [36].

There is a known gap in the literature about primary care-based depression screening for patients with limited English proficiency, best documented in the Spanish-speaking population. None of the used tests were found to be superior, however, fair evidence showed that the Geriatric Depression Scale (GDS) performed best in this population [37,38]. In addition to screening for depression, there is limited research on depression in older adult immigrants. Out of 80,000 studies looking at depression in the immigrant population, only 19 included older adults. Most of these studies did not mention cultural adaptations and its relationship to the presentation of depression. None of the studies examined the role of cultural background and patients’ experience of anxiety or depression. In a collaborative care program of geriatric Latino immigrants, patients used a variety of idioms to describe their experiences with depression, potentially making screening more difficult in this population; bilingual psychotherapists provided the best environment to express patients’ emotions and find solutions to problems [39]. While this may be the best solution in a multicultural immigrant population, this is not always available.

### 3.4. Functional Evaluation

Culturally based functional assessments methods assume familiarity with Western-centric culture. There is a tendency to overclassify impairments in immigrants. Also, not addressed in the Western-centric functional status evaluation are the unique gender and family roles that each culture brings to the functional evaluation [40]. For example, often times, adult children who sponsor their immigrant parents take over instrumental activities of daily living such as financial management, transportation, and shopping. Clinicians inadvertently judge this as a functional impairment. While there are more detailed ways to evaluate function such as the Clinical Dementia Rating [41], they take much longer to administer and are not practical in a clinical setting. Specific scales of function exist for many different cultures, for example, the Everyday Abilities Scale for India [42]. However, it is not practical for providers to know what individual assessment is appropriate for each culture they may be caring for, and this also makes functional assessments less generalizable and difficult to study. 

### 3.5. Advance Care Planning and Decision-Making 

Other important evaluations in the geriatric comprehensive assessment, which requires a more unique approach in a multicultural immigrant population, include beliefs on the use of advance directives, health care decision-making, disclosure and consent, gender issues, and issues related to the end of life [43]. Some cultures favor family- and community-centered health care decision-making over autonomy. Some people believe that bad news hastens death and therefore prefer very little information over truth telling. Some cultures value struggle at the end of life over comfort. This is an area that would benefit from further research. 

### 3.6. Elder Abuse 

Elder abuse and self-neglect are particularly troubling problems for older adults. National studies report a prevalence of 1 in 10 older adults experiencing abuse [44]. These numbers are even higher among minority populations, with one study in a Latino Spanish-speaking community of Los Angeles reporting a prevalence of elder abuse as high as 40% [4]. The use of Spanish-speaking “promotores” in this study, who were surveyors from the community and spoke the language of the study participants, was one of the hypothesized explanations for such high reported rates of elder abuse. The participants may have felt more comfortable disclosing sensitive information to someone whom they felt understood their own culture. This underscores how crucial it is for providers to be culturally sensitive if we are to have any chance at identifying abuse in these populations. Additionally, it is important to recognize that for undocumented immigrant populations, the fear of retaliation or deportation is a barrier for patients to report abuse. 

## 4. Conclusions

In summary, there is a growing need for a more culturally sensitive comprehensive geriatric assessment in order to adequately address the complex social and cultural needs of immigrant older adults. Multicultural geriatric immigrant populations have unique circumstances that can have significant effects on their overall health and wellbeing and are typically not assessed with the traditional comprehensive geriatric assessment. Table 1 summarizes a rubric for best practices in a geriatric immigrant multicultural assessment and identifies gaps in the existing approaches. There is a need for further research and development of assessment tools that can be applied to a multicultural population. 

## Figures and Tables

**Table 1:** Elements of a Multicultural Geriatric Assessment.

**Multicultural Assessment**	**Domain**	**Suggested Assessment Tools or Approach**	**Future Directions**
**1.**	Baseline Preventive Care	Determine prior access to medical care, vaccination status, cancer screening history	Develop consensus guidelines on approach to vaccination assessment, cancer screening in older adult immigrant populations
**2.**	Chronic Conditions	Determine if diagnosis was delayed and address sequelae of untreated illness	
**3.**	Language	Determine literacy level and preferred language	
**4.**	Communication Barriers	Screen for cognitive, hearing, and visual impairment	Develop hearing loss screening assessment that can be used with an interpreter
**5.**	Health Literacy	Determine education level, print literacy, use teach-back method	Enhance low-literacy patient education in multiple languages. Develop and validate training for Community Health Workers (CHWs) on health coaching in older adult immigrant populations.
**6.**	Acculturation Level	Assess self-reported health	Conduct longitudinal studies of self-reported health in older adult immigrants and correlate with health outcomes
**Traditional Geriatric Assessment**	**Domain**	**Suggested Assessment Tools or Approach**	**Future Directions**
**1.**	Cognitive	Rowland Universal Dementia Assessment Scale (RUDAS)	Validate RUDAS in more subpopulations. Develop new low-literacy cognitive screening tools.
**2.**	Delirium	Vigilance testing (e.g., A test), CAM-ICU in preferred language	Develop delirium screening tools for use with interpreters.
**3.**	Mental Health	Geriatric Depression Scale (GDS)	Modify existing tools or develop new culturally specific depression screening tools. Further evaluate outcomes with treatment of depression.
**4.**	Functional Evaluation	Assess the application of change in basic and instrumental activities of daily living, determine cultural expectations for Activities of Daily Living (ADLs) for older adults	Modify existing functional assessment tools to be culturally specific.
**5.**	Advance Care Planning and Decision-Making	Determine culturally defined beliefs regarding health and symptomatology, information sharing, and preferred decision-maker	Further studies are needed on advance care planning in older adult immigrant populations.
**6.**	Elder Abuse and Mistreatment	Determine immigration status (or ask if patient is willing to share documentation status)	Develop culturally sensitive and brief screening tools

## References

[1] Institute of Medicine (US) Committee on the Future Health Care Workforce for Older Americans (2008). Retooling for an Aging America: Building the Health Care Workforce.

[2] U.S. Census (2016). 2015 Summary Files 1,2,3, & 4.

[3] Wiltz T. Aging, Undocumented and Uninsured Immigrants Challenge Cites and States.

[4] DeLiema M., Gassoumis Z.D., Homeier D.C., Wilber K.H. (2012). Determining prevalence and correlates of elder abuse using promotores: Low-income immigrant latinos report high rates of abuse and neglect. J. Am. Geriat. Soc..

[5] Montoya V. (1998). Understanding and combating elder abuse in hispanic communities. J. Elder Abuse Negl..

[6] Xakellis G., Brangman S.A., Hinton W.L., Jones V.Y., Masterman D., Pan C.X., Rivero J., Wallhagen M., Yeo G. (2004). Curricular framework: Core competencies in multicultural geriatric care. J. Am. Geriat. Soc..

[7] Evans K.H., Bereknyei S., Yeo G., Hikoyeda N., Tzuang M., Braddock C.H. (2014). The impact of a faculty development program in health literacy and ethnogeriatrics. Acad. Med..

[8] Velayudhan L., Ryu S.H., Raczek M., Philpot M., Lindesay J., Critchfield M., Livingston G. (2014). Review of brief cognitive tests for patients with suspected dementia. Int. Psychogeriatr..

[9] Jones R.N., Gallo J.J. (2001). Education bias in the mini-mental state examination. Int. Psychogeriatr..

[10] Tombaugh T.N., McIntyre N.J. (1992). The mini-mental state examination: A comprehensive review. J. Am. Geriat. Soc..

[11] Du Y., Xu Q. (2016). Health disparities and delayed health care among older adults in california: A perspective from race, ethnicity, and immigration. Public Health Nurs..

[12] Harris M.F. (2012). Access to preventive care by immigrant populations. BMC Med..

[13] (2013). National Standards for Culturally and Linguistically Appropriate Services (clas) in Health and Health Care.

[14] Chen A.H., Youdelman M.K., Brooks J. (2007). The legal framework for language access in healthcare settings: Title vi and beyond. J. Gen. Intern. Med..

[15] Plejert C., Antelius E., Yazdanpanah M., Nielsen T.R. (2015). There’s a letter called ef’ on challenges and repair in interpreter-mediated tests of cognitive functioning in dementia evaluations: A case study. J. Cross Cult. Gerontol..

[16] Divi C., Koss R.G., Schmaltz S.P., Loeb J.M. (2007). Language proficiency and adverse events in us hospitals: A pilot study. Int. J. Qual. Health Care.

[17] Tamura-Lis W. (2013). Teach-back for quality education and patient safety. Urol. Nurs..

[18] Chesser A.K., Keene Woods N., Smothers K., Rogers N. (2016). Health literacy and older adults: A systematic review. Gerontol. Geriat. Med..

[19] Wolf M.S., Feinglass J., Thompson J., Baker D.W. (2010). In search of ‘low health literacy’: Threshold vs. Gradient effect of literacy on health status and mortality. Soc. Sci. Med..

[20] McDougall G.J., Mackert M., Becker H. (2012). Memory performance, health literacy, and instrumental activities of daily living of community residing older adults. Nurs. Res..

[21] Parker R. (2000). Health literacy: A challenge for american patients and their health care providers. Health Promot. Int..

[22] Berry J.W. (1997). Immigration, acculturation, and adaptation. Appl. Psychol..

[23] Alegria M. (2009). The challenge of acculturation measures: What are we missing? A commentary on thomson & hoffman-goetz. Soc. Sci. Med..

[24] Fayers P.M., Sprangers M.A. (2002). Understanding self-rated health. Lancet.

[25] Lommel L.L., Chen J.L. (2016). The relationship between self-rated health and acculturation in hispanic and asian adult immigrants: A systematic review. J. Immigr. Minor. Health.

[26] TD M. (2016). Perceptions of depression and access to mental health care among latino immigrants: Looking beyond one size fits all. Qual. Health Res..

[27] Kleinman A. (2004). Culture and depression. N. Engl. J. Med..

[28] Kleinman A. (1987). Anthropology and psychiatry. The role of culture in cross-cultural research on illness. Br. J. Psychiatry.

[29] Teut M., Besch F., Witt C.M., Stockigt B. (2019). Perceived outcomes of spiritual healing: Results from a prospective case series. Complement. Med. Res..

[30] Hoff W. (1992). Traditional healers and community health. World Health Forum.

[31] Prince M., Bryce R., Albanese E., Wimo A., Ribeiro W., Ferri C.P. (2013). The global prevalence of dementia: A systematic review and metaanalysis. Alzheimers Dement..

[32] Casas R., Guzmán-Vélez E., Cardona-Rodriguez J., Rodriguez N., Quiñones G., Izaguirre B., Tranel D. (2012). Interpreter-mediated neuropsychological testing of monolingual spanish speakers. Clin. Neuropsychol..

[33] Goudsmit M., van Campen J., Schilt T., Hinnen C., Franzen S., Schmand B. (2018). One size does not fit all: Comparative diagnostic accuracy of the rowland universal dementia assessment scale and the mini mental state examination in a memory clinic population with very low education. Dement. Geriat. Cogn. Dis. Extra.

[34] Meagher J., Leonard M., Donoghue L., O’Regan N., Timmons S., Exton C., Cullen W., Dunne C., Adamis D., Maclullich A.J. (2015). Months backward test: A review of its use in clinical studies. World J. Psychiatry.

[35] Resource Language Translations for Medical Professionals. https://www.icudelirium.org/medical-professionals/downloads/resource-language-translations.

[36] Aranda M.P. (2013). Depression-related disparities among older, low-acculturated U.S. Latinos. Psychiatr. Times.

[37] Reuland D.S., Cherrington A., Watkins G.S., Bradford D.W., Blanco R.A., Gaynes B.N. (2009). Diagnostic accuracy of spanish language depression-screening instruments. Ann. Fam. Med..

[38] Limon F.J., Lamson A.L., Hodgson J., Bowler M., Saeed S. (2016). Screening for depression in latino immigrants: A systematic review of depression screening instruments translated into spanish. J. Immigr. Minor. Health.

[39] Camacho D., Estrada E., Lagomasino I.T., Aranda M.P., Green J. (2018). Descriptions of depression and depression treatment in older hispanic immigrants in a geriatric collaborative care program. Aging Ment. Health.

[40] Pandav R., Fillenbaum G., Ratcliff G., Dodge H., Ganguli M. (2002). Sensitivity and specificity of cognitive and functional screening instruments for dementia: The indo-u.S. Dementia epidemiology study. J. Am. Geriat. Soc..

[41] Morris J.C. (1997). Clinical dementia rating: A reliable and valid diagnostic and staging measure for dementia of the alzheimer type. Int. Psychogeriatr..

[42] Fillenbaum G.G., Chandra V., Ganguli M., Pandav R., Gilby J.E., Seaberg E.C., Belle S., Baker C., Echement D.A., Nath L.M. (1999). Development of an activities of daily living scale to screen for dementia in an illiterate rural older population in India. Age Ageing.

[43] Suurmond J., Seeleman C. (2006). Shared decision-making in an intercultural context. Barriers in the interaction between physicians and immigrant patients. Patient Educ. Couns..

[44] Acierno R., Hernandez M.A., Amstadter A.B., Resnick H.S., Steve K., Muzzy W., Kilpatrick D.G. (2010). Prevalence and correlates of emotional, physical, sexual, and financial abuse and potential neglect in the united states: The national elder mistreatment study. Am. J. Public Health.

